# Flavor Variations in Precious *Tricholoma matsutake* under Different Drying Processes as Detected with HS-SPME-GC-MS

**DOI:** 10.3390/foods13132123

**Published:** 2024-07-03

**Authors:** Fengming Zhang, Bin Lu, Xinhua He, Fuqiang Yu

**Affiliations:** 1 Germplasm Bank of Wild Species & Yunnan Key Laboratory for Fungal Diversity and Green Development, Kunming Institute of Botany, Chinese Academy of Sciences, Kunming 650201, China; zhangfengming@mail.kib.ac.cn (F.Z.); nongxue022@163.com (B.L.); xinhua.he@uwa.edu.au (X.H.); 2 Key Laboratory for Forest Resources Conservation and Utilization in the Southwest Mountains of China, College of Forestry, Southwest Forestry University, Ministry of Education, Kunming 650224, China; 3 College of Resources and Environment, Southwest University, Chongqing 400715, China

**Keywords:** *Tricholoma matsutake*, HS-SPME-GC-MS, volatile organic compounds, drying process

## Abstract

By employing headspace solid-phase microextraction gas chromatography–mass spectrometry (HS-SPME-GC-MS), this study displayed the compositional changes in volatile organic compounds (VOCs) in Tricholoma matsutake samples subjected to hot-air drying (HAD) and vacuum freeze-drying (VFD) processes from their fresh samples. A total of 99 VOCs were detected, including 2 acids, 10 aldehydes, 10 alcohols, 13 esters, 12 ketones, 24 alkanes, 14 olefins, 7 aromatic hydrocarbons, and 7 heterocyclic compounds. Notably, the drying process led to a decrease in most alcohols and aldehydes, but an increase in esters, ketones, acids, alkanes, olefins, aromatic, and heterocyclic compounds. Venn diagram (Venn), principal component analysis (PCA), and partial least squares-discriminant analysis (PLS-DA) analyses enabled an easy and rapid distinction between the VOC profiles of *T. matsutake* subjected to different drying methods. Among the identified VOCs, 30 were designated as marker VOCs indicative of the employed drying process. And the VFD method was more capable of preserving the VOCs of fresh *T. matsutake* samples than the HAD method. Benzaldehyde, 1-Octen-3-ol, 3-Octanol, and (E)-2-Octen-1-ol were identified as markers for FRESH *T. matsutake*. Conversely, (E)-3-Hexene, lavender lactone, and α-Pinene were associated with VFD *T. matsutake*. For HAD *T. matsutake*, olefins, pyrazine, and esters, particularly ocimene, 2,5-Dimethyl-pyrazine, and methyl cinnamate, significantly contributed to its particularities. The results from this present study can provide a practical guidance for the quality and flavor control of volatile organic compounds in preciously fungal fruiting bodies by using drying processes.

## 1. Introduction

*Tricholoma matsutake*, a member of ectomycorrhizal fungus, is a highly valued delicacy for its distinctive flavor and taste. The volatile organic compounds (VOCs) of *T. matsutake* mainly include alcohols, aldehydes, acids, esters, ketones, sulfur compounds, olefins, and C8 aliphatic compounds [1]. The differences of *T. matsutake* VOCs have been investigated under different fried heating/pan-frying temperatures and times [2,3], geographical origins [4,5], grades [6], and cold storage times [7]. Furthermore, the differences of volatile compounds in pileus and stipe [4,8], fresh- and hot-air drying (HAD) [9], and raw and convection-broiler-cooked [10] *T. matsutake* were also observed. C8 compounds (1-Octen-3-ol, 1-Octanol, (E)-2-octen-1-ol, 1-Octen-3-one, 3-Octanol, 3-Octanone, and benzeneacetaldehyde) and methyl cinnamate are the most frequent VOCs of *T. matsutake*. In particular, methyl cinnamate provides the characteristic aroma components of *T. matsutake* samples.

An important factor in determining the quality and flavor of dried *T. matsutake* is closely related to the drying method [9]. Fresh *T. matsutake* has a high water content and is not resistant to storage, making it prone to various physiological changes after being picked. If no protection and anti-corrosion measures are carried out, *T. matsutake* is easily deteriorated, leading to the loss of nutritional components and a decrease in its edible and market value. Therefore, drying is usually an important storage method for *T. matsutake* to effectively control moisture and extend its food shelf life [7]. During the drying and dehydration process, the concentration of C8 compounds is significantly reduced due to the Maillard reaction or the destruction of tissue cells [11]. This process also leads to the formation of some volatile compounds, including hexaldehyde, heptal, 2(5H)-furanone, acetophenone, nonylaldehyde, phenacetaldehyde, etc., resulting in a partial loss of flavor and reduced quality of *T. matsutake*; thus, the selection of different drying methods is of great significance for preserving the flavor of *T. matsutake*.

Due to the limited research on the impact of various drying processes on volatile organic compounds of *T. matsutake*, in this present study, fresh *T. matsutake* was used as the raw material, and dried *T. matsutake* was prepared with hot-air drying (HAD) and vacuum freeze-drying (VFD) processes. Headspace solid-phase microextraction (HS-SPME) technology, known for its high selectivity, enrichment efficiency, and rapid analysis capabilities, was employed for extraction and analysis to assess the impact of various drying methods on *T. matsutake* flavor. The aim of this study is to provide a theoretical reference for the further process and utilization of *T. matsutake* and a practical basis for promoting its commercial application.

## 2. Materials and Methods

### 2.1. Fungal Materials and Sample Production

**Figure 1:**
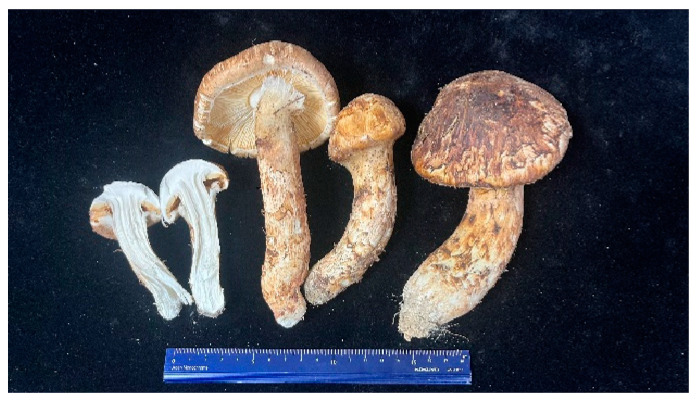
The fruiting bodies of *Tricholoma matsutake*.

*T. matsutake* samples were obtained from the Diqing Tibetan Autonomous Prefecture, Yunnan, Southwest China. FRESH samples were stored at room temperature; VFD (vacuum freeze drying, FD-1A-50, BIOCOOL, Beijing, China) samples were frozen at −80 °C for 24 h, and then vacuum freeze-dried for 24 h at −40 to −50 °C, vacuum 10 Pa; HAD (hot air drying, GZX-GF 101-3 BS, Yuejin Medical Co., Shanghai, China) samples were weighed at a certain quantity, cut into uniform slices, dried for 4 h at 60 to 70 °C, and then dried for 2 h at 70 to 100 °C (Figure 1).

### 2.2. Headspace Solid-Phase Microextraction (HS-SPME)

Samples were extracted using a manual headspace sampling system, equipped with a 50/30 µm DVB/CAR/PDM fiber (Supelco, Bellefonte, PA, USA). Fresh samples were chopped and weighed at 5.0 g. The samples were placed in 40 mL headspace vials, pre-equilibrated at 45 °C for 5 min, and then extracted for 40 min at the same temperature. After extraction, the fiber was immediately inserted into the injection port of GC-MS for thermal desorption at 250 °C for 10 min.

### 2.3. Gas Chromatography–Mass Spectrometry (GC-MS) Analysis

Gas chromatography–mass spectrometry (7890A-5975C, Agilent, Santa Clara, CA, USA) with a capillary column DB-5MS (30 m × 0.25 mm, 0.25 μm, Agilent, USA) was utilized. The injection port was operated in splitless mode at 250 °C. The following chromatographic separations were performed: 40 °C held for 5 min, increased to 180 °C at a rate of 5 °C/min and held for 2 min, and then to 260 °C at a rate of 10 °C/min. Helium was used as the carrier gas with a flow rate of 1.0 mL/min. The operating conditions for the MS system were as follows: the ion source was set at 230 °C, and the electron ionization mode was at 70 eV with mass ranges from 35 to 500 *m*/*z*.

### 2.4. Statistical Analysis

Each component underwent NIST11 library search, and data were analyzed using MSD ChemStation software (Agilent Technologies, version G1701EA E. 02. 02. 1431). For each analyte, its relative mass fraction was calculated by the peak area normalization method.

Data were reported as means ± standard deviation (SD). There were three replicates for each treatment, and *p*-values for differences between different treatments within the same species were examined using Student’s *t*-test (*p* ≤ 0.05). Principal component analysis (PCA) and partial least squares–discriminant analysis (PLS-DA) were performed using Simca-p 14.1 software, while Origin 2021 was utilized for correlation analysis and visualization of any differences between samples.

## 3. Results

### 3.1. Changes in HS-SPME-GC-MS of T. matsutake with Differing Drying Processes

**Figure 2:**
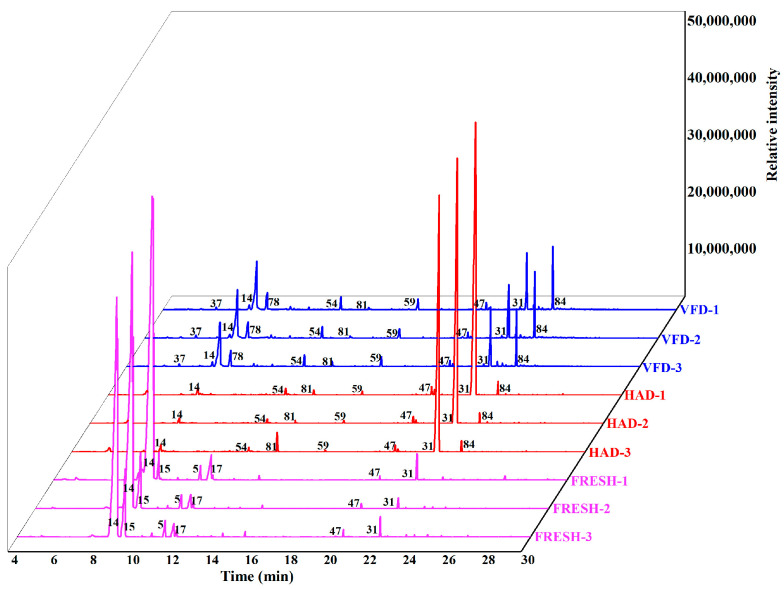
Total ion chromatograms of *T. matsutake* during the drying process. (Number of peaks is shown in Table 1).

The varying VOCs in *T. matsutake* with differing drying processes were analyzed using HS-SPME-GC-MS. The signals in the 20–25 min retention time were rapidly increased in VFD and HAD while remaining consistent among the ion chromatograms of the other samples (Figure 2). A mass of VOCs was indicated to be released during the drying processes. Herein, a total of 99 different volatile compounds were identified by the HS-SPME-GC-MS analysis, including 2 acids, 10 aldehydes, 10 alcohols, 13 esters, 12 ketones, 24 alkanes, 14 olefins, 7 aromatic hydrocarbons, and 7 heterocyclic compounds (Table 1). The relative amount of each compound was obtained by its peak area normalization.

### 3.2. Variation in VOCs of T. matsutake

#### 3.2.1. Changes in the Types and Relative Contents of VOCs

**Figure 3:**
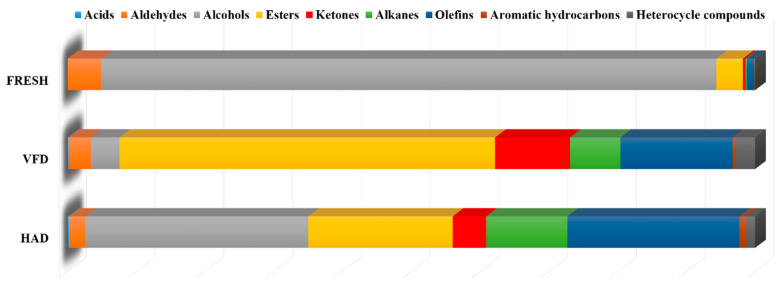
Classification analysis of VOCs in dry *T. matsutake* after different drying processes.

A total of 66 VOCs were detected in the VFD group, with a high relative content of 36.82% hydrocarbons (alkanes and olefins), 32.38% alcohols, and 21.01% esters, respectively. In the HAD group, a total of 65 volatile components were detected, with 54.60% esters, 23.67% hydrocarbons, and 10.89% ketones, respectively. In the FRESH group, a total of 44 VOCs were detected, with 89.47% alcohols, but without acid substances (Table 1 and Figure 3). Different drying methods had a significant effect on the VOCs in *T. matsutake*. After drying, the relative contents of volatile components in *T. matsutake* increased, while both alcohols and aldehydes were decreased. However, the relative content of other components, such as esters, increased. Esters were formed by the interaction of alcohols and free fatty acids resulting from fat oxidation [12].

#### 3.2.2. Analysis of Major VOCs in Different-Drying *T. matsutake*

The main VOCs of the FRESH group were 1-Octen-3-ol (68.67%), 3-Octanol (16.38%), (E)-2-Octen-1-ol (3.33%), methyl cinnamate (2.07%), and benzaldehyde (1.16%). The main volatile components of the VFD group were 1-Octen-3-ol (31.41%), methyl cinnamate (19.93%), β-Barbatene (15.02%), (E)-3-Hexene (6.65%), dodecane (4.14%), undecane (3.75%), 1-Octen-3-one (1.95%), octane (1.45%), and 5-Ethenyldihydro-5-methyl-2(3H)-furanone (1.08%). The main VOCs of the HAD group were methyl cinnamate (52.76%), β-Barbatene (7.64%), 2(5H)-Furanone (6.38%), ocimene (5.34%), dodecane (3.80%), 2,5-Dimethylpyrazine (2.63%), benzaldehyde (1.85%), and octane (1.07%). Methyl cinnamate was the most important volatile component of *T. matsutake*. The relative content of methyl cinnamate was the highest at 52.76% in the HAD group, followed by 19.93% in the VFD group and 2.07% in the FRESH group. 1-Octen-3-ol was the major common VOC in the FRESH and VFD groups, comprising 68.67% and 31.41%, respectively (Table 1).

#### 3.2.3. Analysis of the Unique and Common VOCs in Different Drying Processes of *T. matsutake*

**Figure 4:**
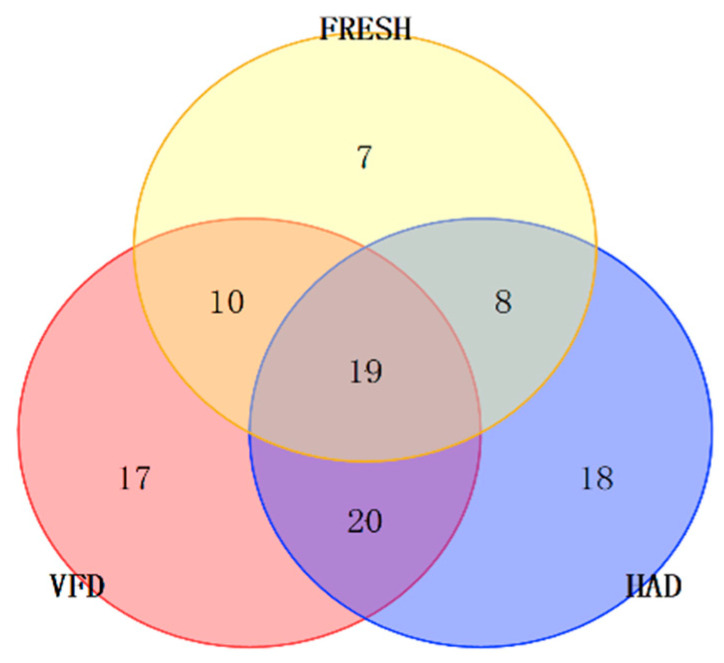
The Venn diagram of VOCs.

From Table 2 and Figure 4, the VFD, HAD, and FRESH groups of *T. matsutake* contained 17, 18, and 7 unique components, with respective relative contents of 7.2%, 4.49%, and 0.58%. The VFD group was divided into seven alkanes, four aromatics, two heterocyclics, and one of each acid, aldehyde, ester and olefin. The HAD group was divided into five alkanes, three heterocytypes, two kinds of alcohol, ketone and olefin, and one kind of acid, aldehyde, ester and aromatic. And the FRESH group was divided into three aldehydes and one of alcohol, ester, ketone and alkanes. These three groups had 19 common components, with relative contents of 73.53% (VFD), 69.19% (HAD), and 80.95% (FRESH).

### 3.3. Characteristic VOCs via PCA and PLS-DA

**Figure 5:**
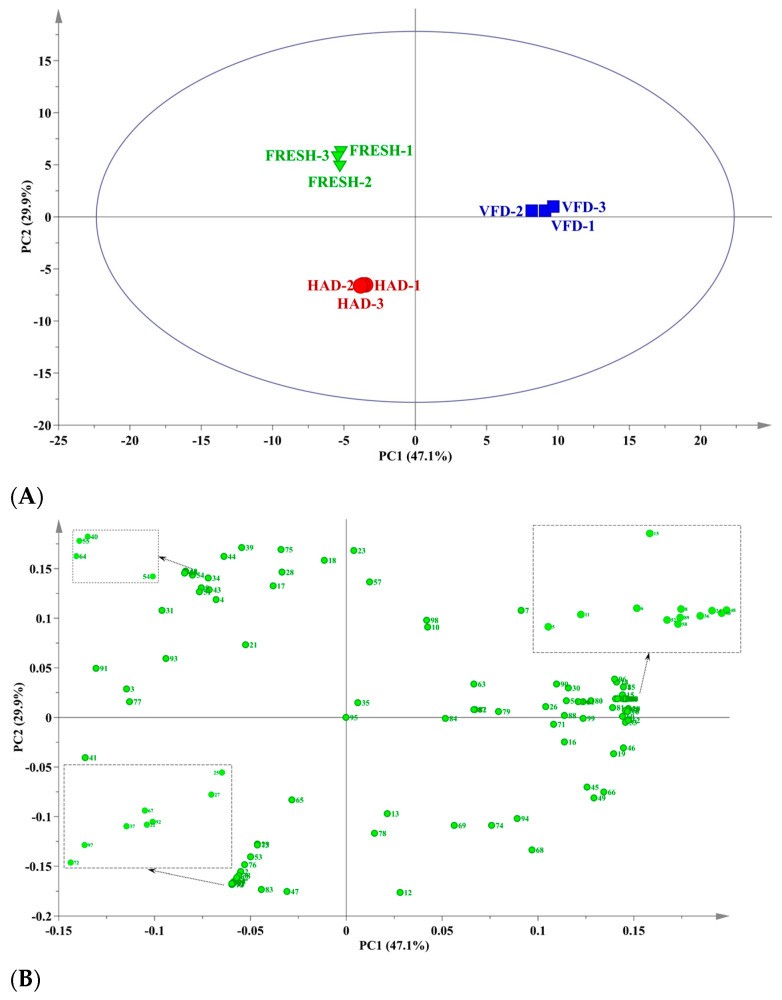

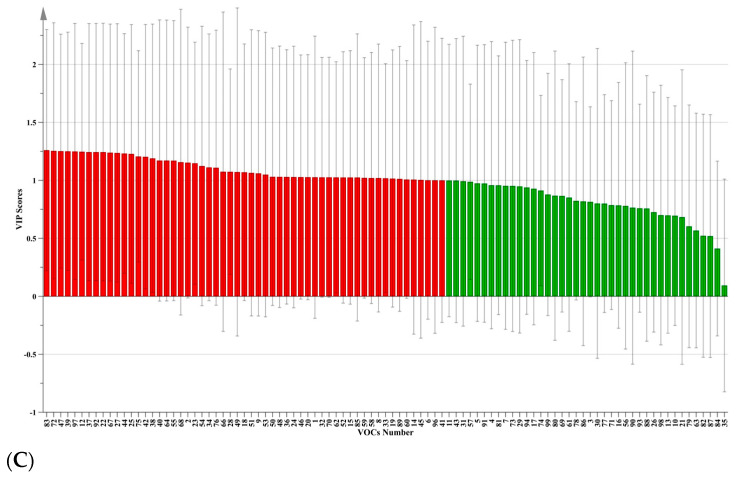
PCA and PLS-DA analysis of VOCs of dry *T. matsutake* after in different drying methods. (**A**) PCA analysis of VOCs of *T. matsutake* after different drying treatments. (**B**) PCA loading diagram. (Numbers in the figure are the same as those in Table 1). (**C**) VIP score diagram of PLS-DA model. (Numbers in the figure are the same as those in Table 1).

To further understand the differences in the VOCs under three key *T. matsutake* processing points, a total of 99 significantly different volatiles among samples were used to run the PCA (Figure 5A). PC1 and PC2 were the qualitative and quantitative analysis of VOCs in the spectrum, with contribution rates of 47.1% and 29.9%, respectively. There was no overlap among the three sample groups in Figure 5A, thereby indicating significant differences in the VOCs among the sample groups. The samples with different drying methods can thus be well distinguished by PCA. The value of PC2 was increased in the following order: HAD < VFD < FRESH. A large separation between HAD and the other drying groups implied significant changes in the VOCs caused by the HAD method. Conversely, the smaller separation between FRESH and VFD indicated that the effects of drying treatments on their chemical profiles were similar. Therefore, the results revealed that HS-SPME-GC-MS coupled with PCA can rapidly distinguish *T. matsutake* via different drying treatments, and thus be a promising quality control method of the three processes. In Figure 5B, the differentiation among the *T. matsutake* samples from three drying methods was more clearly demonstrated through score plots combined with loading plots, effectively illustrating the correlations between the 99 VOCs and the samples.

As show in Figure 5B, the VFD samples were predominantly located in the I quadrant, with the VFD samples being characterized by (E)-2-Octenal (5), atropaldehyde (6), decanal (8), (E,E)-2,4-Decadienal (11), 3-Octanol (15), γ-Valerolactone (24), 2(5H)-Furanone (36), 2-Cyclopropyl-butane (48), 3-Methyl-nonane (50), 4,7-Dimethyl-undecane (52), methyl-cyclooctane (58), and naphthalene (89). The FRESH samples were scattered in quadrant II, and positively corrected with benzeneacetaldehyde (4), (E,E)-2,4-nonadienal (9), diisobutyl phthalate (34), 2,2,5-Trimethyl-3-hexanone (40), acetophenone (43), 2-Methyl-pentane (51), undecane (54), pentyl-cyclohexane (55), and 3-Methyl-dodecane (64). The HAD samples (in the III quadrant) were more correlated to butanoic acid (2), nerolidol (22), tetrahydrofurfuryl propionate (25), caprylic acid methyl ester (27), 2-Ethylhexyl hexyl sulfite (29), 1-Octen-3-one (37), 1-Hepten-3-one (38), 3-Octen-2-one (42), 1-Ethyl-1-methyl-cyclopentane (53), tetradecane (67), 1,3-Octadiene (72), 2,4-Dimethyl-1-heptene (73), α-Pinene (76), 2,4-Di-tert-butylphenol (92), and 1,1-Dioxide-2-methylthiolane (97).

Upon identifying a classification trend among the three matsutake samples via PCA, a further examination into the variables driving these differences was conducted through discriminant analysis using PLS-DA. The VIP score quantified the influence of different compounds in the PLS-DA model, with higher VIP values signifying a greater contribution to the classification of *T. matsutake* across the three treatments. The VIP score map from the PLS-DA model (Figure 5C) identified 57 volatile substances as key differential components capable of distinguishing the three processing methods of *T. matsutake* (VIP > 1, *p* < 0.05), including butanoic acid (2), nerolidol (22), tetrahydrofurfuryl propionate (25), caprylic acid methyl ester (27), methyl oct-2-enoate (28), diisobutyl phthalate (34), 1-Octen-3-one (37), 3-Octanone (39), 2,2,5-Trimethyl-3-hexanone (40), 3-Octen-2-one (42), pentyl-cyclohexane (55), 2,6,10-Trimethyl-dodecane (66), (+)-Sativen (83), 1,1-Dioxide-2-methylthiolane (97), etc. This analysis, along with Table 1, highlights significant variations in the relative contents of these VOCs across the matsutake samples, underlining their importance in defining the unique aroma profiles of each sample.

## 4. Discussion

Differences were found in the types and contents of VOCs of *T. matsutake* compared with a previous study. These variances could be attributed to differences in the fried heating/pan-frying temperatures and times [2,3], cold storage times [7], and geographical origins [4,5,13], etc.

The VOCs mostly originate from the chemical or enzymatic oxidation of unsaturated fatty acids, followed by interactions with proteins, peptides, and free amino acids. Other volatile compounds result from the Strecker degradation of free amino acids and Maillard reactions [14].

The contents of C8 compounds, especially 1-octen-3-ol, 3-octanone, 1-octanol, 3-octanol, and (E)-2-octen-1-ol, were significantly decreased after the drying process. Yang et al. [15] reported the degradation of C8 components during heat treatment. Due to the destruction of the cell wall and cytoplasm, more various intracellular components are released from cells and participate in the reaction during drying while some VOCs might be formed.

Aldehydes significantly contribute to the formation of flavors in edible fungi, characterized by their abundant presence and relatively low odor thresholds [16]. Our analysis revealed that aldehydes varied in quantity across the samples, with five compounds identified in both VFD and HAD samples, and eight compounds in the FRESH samples. Notably, aldehydes constituted the highest proportion of volatile compounds in the FRESH sample, accounting for up to 4.90% of the relative content. Similarly, substantial quantities of aldehydes were detected in the other two mushroom samples. All identified aldehyde compounds were classified as unsaturated aldehydes, commonly recognized as the oxidation products of unsaturated fatty acids [17]. The variation in aldehyde content and composition across samples was primarily attributed to the different drying methods employed. In the context of actual production, processing conditions, particularly drying methods, significantly influence the concentration of aldehydes [18]. Contrary to expectations, the quantity of aldehydes in our study exhibited a decrease, highlighting the impact of processing techniques on aldehyde profiles.

Alcohols are primarily formed through the lipid oxidation of polyunsaturated fatty acids, which typically have a higher threshold and contribute to a soft and sweet aroma [13]. In the VFD, HAD, and FRESH samples, 7, 8, and 5 alcohols were observed, respectively. Simultaneously, alcohols were the most abundant group in the FRESH sample, occupying 89.47% of the total peak area. In addition to fatty alcohols, terpenes such as linalool and nerolidol were also included, which were detected in the samples. Among these samples, the sample from the FRESH group contained the highest alcohol content (89.47% at room temperature), which decreased with increasing drying temperatures: VFD (32.38% at −40 °C to −50 °C for 24 h) and HAD (4.13% at 60 °C to 70 °C for 4 h; 70 °C to 100 °C for 2 h). These results were in accordance with other reports that focused on the change in alcohols in edible fungi at different heating temperatures. Additionally, (E)-2-octen-1-ol, 1-octene-3-ol, and 1-octanol were detected in all the three *T. matsutake* samples. The alcohol content was decreased after the drying processes. It could contribute to the development of matsutake mushroom flavor. The 1-octene-3-ol alcohol group was identified as the major volatile compound found in raw mature *T. matsutake*. This aliphatic unsaturated alcohol is beneficial for enhancing the manifestation of mushroom flavor. 1-Octen-3-ol, with a typical odor reminiscent of mushrooms, lavender, rose and hay, has been detected and reported in most edible fungi [6,19]. 1-octen-3-ol is the product of autoxidation and/or the enzymatic oxidation and cleavage of linoleic acid in mushroom [20]. Alcohols are a class of VOCs that have been detected in raw mushroom and mushroom products like *Volvariella volvacea*, shiitake mushrooms, and *Agaricus bisporus* [21].

Esters mainly exist in fruits and exhibit a sweet and fruity flavor, which is associated with the oxidation of unsaturated fatty acids [22]. The content of esters varied greatly in *T. matsutake* samples at different drying temperatures. The total content of esters was increased with the increased in drying temperature, ranging from 3.85% to 54.60% in samples dried using FRESH, VFD, and HAD methods. Processing methods, such as drying, could lead to an increase in esters [18]. Among these esters, the contents of tetrahydrofurfuryl propionate and methyl cinnamate in dried samples (VFD and HAD) was higher than these in the FRESH sample. Tetrahydrofurfuryl propionate imparts a fruit aroma, while methyl cinnamate impacts a strawberry aroma.

A lower content of ketones was found in *T. matsutake*, accounting for 0.40–10.89% of the total VOCs. The content of ketones showed a relative increasing trend with the increase in drying temperature. Several ketones were generated during the drying process as a result of the thermal degradation of amino acids or the thermal oxidation of polyunsaturated fatty acids, such as leucine, phenylalanine, and threonine [23,24]. Different amounts of ketones were detected, with 7, 11, and 5 compounds identified in the VFD, HAD, and FRESH samples, respectively. Ketones were observed to be the most abundant compounds in HAD samples, accounting for 10.89% of the peak area, while in other samples, they were relatively low. Ketones are products of the decomposition of esters or the oxidation of alcohols [25].

Hydrocarbons (alkanes and olefins) are generally not considered to have an aroma contribution due to their relatively high odor threshold. However, alkanes could help enhance the flavor of food [15]. The hydrocarbons content was increased at both low and high drying temperatures. The VFD samples (low temperature) showed a more significant increase compared to the HAD samples (high temperature), with contents of 36.82% and 23.67% of hydrocarbons, respectively. This phenomenon can be attributed to the dynamic equilibrium between the cracking reaction of alkoxyl radicals and the loss of volatile components at higher temperatures [25]. Some alkanes and olefins were also detected in the samples, accounting for 15, 26, and 25 compounds in the FRESH, VFD, and HAD samples, respectively.

All samples also contained a very small number of aromatic hydrocarbons, which were identified as six compounds in VFD, two compounds in HAD, and one compound in FRESH samples. A minimal number of acid compounds was only detected in VFD (1 compound, 0.23%) and HAD (1 compound, 0.18%) samples, but not detectable in the FRESH samples. These acid compounds and aromatic hydrocarbons have not been reported in *T. matsutake* by other references [8]. Eleven acids were identified from different geographical origins of *T. matsutake* [24], which differed from our results.

Heterocyclic compounds, especially pyrazine, are an important source of the unique VOCs of *T. matsutake*, which has a strong odor intensity with nutty and roasted flavors [10]. It was observed that 2,5-Dimethyl pyrazine changed with the increase in drying temperature for the VFD (0.11%) and HAD (2.63%) samples.

By analyzing the relative contents of common VOCs in different drying methods (FRESH (80.95%) > VFD (73.53%) > HAD (69.19%), we can see that the VFD samples were relatively close to the FRESH samples. As seen from the Venn diagram of the VOCs, the VFD samples had a greater amount of common VOCs with the FRESH samples compared to the HAD samples. (E)-2-octenal, 1-Octen-3-ol, 1-Octen-3-one, 3-Octanol and 3-Octen-2-one had a characteristic mushroom-like odor and were recognized as a key odorant in forming the distinctive mushroom aroma, playing a role in the reconciliation and complementation of the flavor of *T. matsutake* samples [24]. And they were much better retained in the VFD than the HAD sample. Especially, the relative amount of 1-Octen-3-ol and 1-Octen-3-one in VFD samples (31.41%, 1.95%) were much more abundant than HAD samples (0.77%, 0.72%). Moreover, the smaller separation between the FRESH and VFD samples indicated that the effects of drying treatments on their chemical profiles were similar. Thus, the VFD method was more capable of preserving the VOCs of fresh *T. matsutake* samples than the HAD method.

## 5. Conclusions

This study focused on the diversity of the VOCs in *T. matsutake* samples under fresh, hot-air-drying, and vacuum freeze-drying treatments. SPME-GC-MS was successfully employed to identify flavor compounds formed after different drying treatments of *T. matsutake*. A total of 99 flavor substances were identified across these three treatments of *T. matsutake*, including acids, aldehydes, alcohols, ketones, esters, alkanes, olefins, aromatic hydrocarbons and heterocycle compounds. In addition, the PCA analysis from GC-MS showed that samples from different drying processes could be distinguished by their VOCs, and the VFD method was more capable of preserving the VOCs of fresh *T. matsutake* samples than the HAD method. Based on the VIP score diagram of the PLS-DA model on VOCs in these samples, 30 VOCs were identified as potentially contributing to the aroma of *T. matsutake*. Benzaldehyde, 1-Octen-3-ol, 3-Octanol, and (E)-2-Octen-1-ol were identified as the primary compounds responsible for the mushroom aromas of fresh *T. matsutake* before the drying process. This study demonstrated the potential of HS-SPME-GC-IMS in combination with PCA and PLS-DA as a reliable analytical screening technique to quickly and sensitively identify and classify the VOCs of *T. matsutake*. Results from this present study can provide a theoretical and practical basis for the quality control of flavor in the processing of preciously edible fungal products.

## Figures and Tables

**Table 1:** The relative amounts of volatile compounds in different-drying *T. matsutake*.

No.	CAS	Compound	Formula	MW	RT [min]	Relative Amount %		
						VFD	HAD	FRESH
		Acids						
1	503-74-2	3-Methyl-butanoic acid	C_5_H_10_O_2_	102.1	5.52	0.23	nd	nd
2	107-92-6	Butanoic acid	C_4_H_8_O_2_	88.1	9.40	nd	0.18	nd
		Aldehydes						
3	100-52-7	Benzaldehyde	C_7_H_6_O	106.1	7.72	0.87	1.85	1.16
4	122-78-1	Benzeneacetaldehyde	C_8_H_8_O	120.1	10.79	0.12	0.86	0.57
5	2548-87-0	(E)-2-Octenal	C_8_H_14_O	126.2	11.45	0.74	0.16	2.80
6	4432-63-7	Atropaldehyde	C_9_H_8_O	132.1	14.94	nd	nd	0.06
7	18829-56-6	trans-2-Nonenal	C_9_H_16_O	140.2	15.16	nd	nd	0.05
8	112-31-2	Decanal	C_10_H_20_O	156.2	17.16	0.02	nd	0.05
9	5910-87-2	(E,E)-2,4-Nonadienal	C_9_H_14_O	138.2	17.49	nd	nd	0.12
10	4411-89-6	2-Phenylbut-2-enal	C_10_H_10_O	146.1	19.33	nd	0.19	nd
11	25152-84-5	(E,E)-2,4-Decadienal	C_10_H_16_O	152.2	20.82	nd	0.21	0.09
12	13019-16-4	2-Butyl-2-octenal	C_12_H_22_O	152.2	22.09	0.65	nd	
		Alcohols						
13	111-27-3	1-Hexanol	C_6_H_14_O	102.1	4.74	nd	nd	0.15
14	3391-86-4	1-Octen-3-ol	C_8_H_16_O	128.2	8.79	31.41	0.77	68.67
15	589-98-0	3-Octanol	C_8_H_18_O	130.2	9.48	0.06	nd	16.38
16	18185-81-4	3-Octen-1-ol	C_8_H_16_O	128.2	11.77	nd	0.59	nd
17	18409-17-1	(E)-2-Octen-1-ol	C_8_H_16_O	128.2	11.97	0.28	0.44	3.33
18	111-87-5	1-Octanol	C_8_H_18_O	130.2	12.05	0.02	0.75	0.94
19	34995-77-2	trans-Furan linalool oxide	C_10_H_18_O_2_	170.2	12.45	0.25	0.73	nd
20	818-81-5	2-Methyl-1-octanol	C_9_H_20_O	144.2	12.65	0.29	0.50	nd
21	3913-02-8	2-Butyl-1-octanol	C_12_H_26_O	186.3	20.35	nd	0.07	nd
22	40716-66-3	Nerolidol	C_15_H_26_O	222.3	25.61	0.07	0.28	nd
		Esters						
23	106-70-7	Methyl hexanoate	C_7_H_14_O_2_	130.1	6.43	0.11	nd	0.01
24	108-29-2	γ-Valerolactone	C_5_H_8_O_2_	100.1	7.34	0.24	0.25	nd
25	637-65-0	Tetrahydrofurfuryl propionate	C_8_H_14_O_3_	158.2	10.85	0.10	0.16	0.07
26	695-06-7	5-Ethyloxolan-2-one	C_6_H_10_O_2_	114.1	11.08	0.10	nd	nd
27	111-11-5	Caprylic acid methyl ester	C_9_H_18_O_2_	158.2	13.85	nd	nd	0.17
28	7367-81-9	Methyl oct-2-enoate	C_9_H_16_O_2_	156.2	15.59	0.32	0.27	0.74
29	959067-41-5	2-Ethylhexyl hexyl sulfite	C_14_H_30_O_3_S	278.4	20.24	nd	0.10	nd
30	1191-02-2	Methyl dec-4-enoate	C_11_H_20_O_2_	184.2	20.51	0.16	nd	0.54
31	103-26-4	Methyl cinnamate	C_10_H_10_O_2_	162.2	22.45	19.93	52.76	2.07
32	4493-42-9	Methyl 2E,4Z-decadienoate	C_11_H_18_O_2_	182.2	22.62	nd	0.43	0.06
33	79837-88-0	Methyl (Z)-dodec-5-enoate	C_13_H_24_O_2_	212.3	24.80	nd	0.54	0.18
34	84-69-5	Diisobutyl phthalate	C_16_H_22_O_4_	278.3	30.10	0.02	nd	0.01
35	84-74-2	Dibutyl phthalate	C_16_H_22_O_4_	278.3	31.67	0.03	0.09	nd
		Ketones						
36	497-23-4	2(5H)-Furanone	C_4_H_4_O_2_	84.0	5.97	0.75	6.38	nd
37	4312-99-6	1-Octen-3-one	C_8_H_14_O	126.1	8.36	1.95	0.72	nd
38	2918-13-0	1-Hepten-3-one	C_7_H_12_O	112.1	8.54	nd	0.14	0.24
39	106-68-3	3-Octanone	C_8_H_16_O	128.2	8.66	nd	0.84	nd
40	14705-50-1	2,2,5-Trimethyl-3-hexanone	C_9_H_18_O	142.2	9.20	nd	0.35	nd
41	1073-11-6	Lavender lactone	C_7_H_10_O_2_	126.1	10.46	1.08	0.66	nd
42	18402-82-9	3-Octen-2-one	C_8_H_14_O	126.2	10.64	0.46	0.19	nd
43	98-86-2	Acetophenone	C_8_H_8_O	120.1	11.67	nd	nd	0.02
44	3508-78-9	3-Allylpentane-2,4-dione	C_8_H_12_O_2_	140.1	11.74	nd	0.38	0.05
45	693-54-9	2-Decanone	C_10_H_20_O	156.2	16.45	0.29	0.26	0.01
46	927-49-1	6-Undecanone	C_11_H_22_O	170.2	19.47	0.08	0.44	nd
47	112-12-9	2-Undecanone	C_11_H_22_O	170.3	20.13	0.26	0.53	0.08
		Alkanes						
48	5750-02-7	2-Cyclopropyl-butane	C_7_H_14_	98.2	4.14	nd	nd	0.01
49	2415-72-7	Propyl-cyclopropane	C_6_H_12_	84.1	4.65	0.23	nd	nd
50	5911-04-6	3-Methyl-nonane	C_10_H_22_	142.2	8.12	0.04	0.09	nd
51	107-83-5	2-Methyl-pentane	C_6_H_14_	86.1	9.61	nd	0.04	nd
52	17301-32-5	4,7-Dimethyl-undecane	C_13_H_28_	184.3	11.25	nd	0.45	nd
53	16747-50-5	1-Ethyl-1-methyl-cyclopentane	C_8_H_16_	112.2	11.36	nd	0.21	nd
54	1120-21-4	Undecane	C_11_H_24_	156.3	13.02	3.75	nd	nd
55	4292-92-6	Pentyl-cyclohexane	C_11_H_22_	154.2	14.21	0.05	nd	nd
56	71138-64-2	3-Methyl-undecane	C_12_H_26_	168.3	14.52	nd	0.14	nd
57	62238-12-4	2,3,6-Trimethyl-decane	C_13_H_28_	184.3	15.36	0.03	nd	nd
58	1502-38-1	Methyl-cyclooctane	C_9_H_18_	126.2	16.46	0.25	nd	nd
59	112-40-3	Dodecane	C_12_H_26_	170.3	16.88	4.14	3.80	nd
60	560-21-4	2,3,3-Trimethyl-pentane	C_8_H_18_	114.2	19.21	0.01	0.05	nd
61	5881-17-4	3-Ethyl-octane	C_10_H_22_	142.2	19.58	0.02	0.63	nd
62	111-65-9	Octane	C_8_H_18_	114.2	20.39	1.45	1.07	0.01
63	17301-33-6	4,8-Dimethyl-undecane	C_13_H_28_	184.3	21.37	nd	0.28	nd
64	17312-57-1	3-Methyl-dodecane	C_8_H_18_	184.3	21.83	0.04	0.07	nd
65	1072-16-8	2,7-Dimethyl-octane	C_10_H_22_	142.2	22.11	0.24	0.08	0.01
66	3891-98-3	2,6,10-Trimethyl-dodecane	C_15_H_32_	212.4	22.24	0.09	nd	nd
67	629-59-4	Tetradecane	C_14_H_30_	198.3	22.79	0.96	0.32	0.11
68	14905-56-7	2,6,10-Trimethyltetradecane	C_17_H_36_	240.4	23.97	0.25	nd	nd
69	629-62-9	Pentadecane	C_15_H_32_	212.4	24.71	0.17	nd	0.01
70	17302-01-1	3-Ethyl-3-methylheptane	C_10_H_22_	142.2	25.27	nd	0.05	0.01
71	563-16-6	3,3-Dimethyl-hexane	C_8_H_18_	114.2	26.38	0.09	0.07	0.01
		Olefins						
72	1002-33-1	1,3-Octadiene	C_8_H_14_	110.1	3.29	0.63	nd	0.03
73	19549-87-2	2,4-Dimethyl-1-heptene	C_9_H_18_	126.2	3.46	nd	0.39	nd
74	694-87-1	Benzocyclobutene	C_8_H_8_	104.1	5.21	0.18	nd	0.48
75	16746-86-4	2,3-Dimethyl-1-hexene	C_8_H_16_	112.2	3.63	nd	0.08	0.04
76	80-56-8	α-Pinene	C_10_H_16_	136.2	6.64	1.15	0.21	0.03
77	690-92-6	(3Z)-2,2-Dimethyl-3-hexene	C_8_H_16_	112.2	7.60	0.10	0.33	0.12
78	13269-52-8	(E)-3-Hexene	C_6_H_12_	84.1	9.31	6.65	0.16	nd
79	61142-36-7	3-Ethyl-2-methyl-1,3-hexadiene	C_9_H_16_	124.2	10.38	nd	0.69	0.18
80	13877-91-3	Ocimene	C_10_H_16_	136.2	13.00	0.23	5.43	nd
81	56728-10-0	3,4,5-Trimethyl-1-hexene	C_9_H_18_	126.2	13.46	0.18	0.66	0.01
82	71138-64-2	3-Methylene-undecane	C_12_H_24_	168.3	16.15	0.12	0.08	nd
83	3650-28-0	(+)-Sativen	C_15_H_24_	204.3	23.05	0.75	nd	nd
84	39863-73-5	(±)-β-Barbatene	C_15_H_24_	204.3	23.77	15.02	7.64	0.21
85	30364-38-6	Dehydro-ar-ionene	C_15_H_22_O	172.2	27.04	nd	0.64	nd
		Aromatic hydrocarbons						
86	100-41-4	Ethylbenzene	C_8_H_10_	106.1	4.26	0.04	nd	nd
87	95-47-6	o-Xylene	C_8_H_10_	106.1	4.54	0.24	nd	0.07
88	99-87-6	p-Cymene	C_10_H_14_	134.2	13.57	0.10	nd	nd
89	91-20-3	Naphthalene	C_10_H_8_	128.1	15.96	0.23	nd	nd
90	90-12-0	1-Methyl-naphthalene	C_11_H_10_	142.1	20.09	0.34	0.16	nd
91	575-43-9	1,6-Dimethyl-naphthalene	C_12_H_12_	156.2	23.12	0.06	nd	nd
92	96-76-4	2,4-Di-tert-butylphenol	C_14_H_22_O	206.3	24.82	nd	0.22	nd
		Heterocycle compounds						
93	109-08-0	Methyl-pyrazine	C_5_H_6_N_2_	94.1	4.39	0.03	nd	nd
94	123-32-0	2,5-Dimethyl-pyrazine	C_6_H_8_N_2_	108.1	5.99	0.11	2.63	nd
95	3777-69-3	2-Pentyl-furan	C_9_H_14_O	138.2	8.84	0.36	nd	nd
95	100-84-5	3-Methylanisole	C_8_H_10_O	122.1	9.97	0.78	nd	0.04
97	1003-46-9	1,1-Dioxide-2-methylthiolane	C_5_H_10_O_2_S	134.1	18.48	nd	0.07	nd
98	111150-30-2	3,5-Dimethyl-2-(3-methylbutyl)pyrazine	C_11_H_18_N2	178.2	20.64	nd	0.16	nd
99	113604-56-1	1,2,3-Trimethyldiaziridine	C_4_H_10_N_2_	86.1	25.90	nd	0.02	nd

Notes: nd = not detected.

**Table 2:** The unique and common VOCs in dry *T. matsutake* after different drying treatments.

Unique VOCs			Common VOCs
VFD	HAD	FRESH	VFD/HAD/FRESH
Ethylbenzene	2,4-Dimethyl-1-heptene	2-Cyclopropyl-butane	α-Pinene
2-Methylpyrazine	3-Octanone	1-Hexanol	(3Z)-2,2-Dimethyl-3-hexene
Propyl cyclopropane	2,2,5-Trimethyl-3-hexanone	Acetophenone	Benzaldehyde
3-Methyl-butanoic acid	Butanoic acid	Caprylic acid methyl ester	Benzeneacetaldehyde
2-Pentyl-furan	2-Methyl-pentane	Atropaldehyde	Tetrahydrofurfuryl propionate
Lavender lactone	4,7-Dimethyl-undecane	trans-2-Nonenal	(E)-2-Octenal
Undecane	1-Ethyl-1-methyl-cyclopentane	(E,E)-2,4-Nonadienal	(E)-2-Octen-1-ol
*p*-Cymene	3-Octen-1-ol		1-Octanol
Pentyl-cyclohexane	3-Methyl-undecane		3,4,5-Trimethylhexene
2,3,6-Trimethyl-decade	1,1-Dioxide-2-methylthiolane		(E)-2-Octenoic acid, methyl ester
Naphthalene	2-Phenylbut-2-enal		2-Decanone
Methyl-cyclooctane	2-Ethylhexyl hexyl sulfite		2-Undecanone
2-Butyl-2-octenal	2-Butyl-1-octanol		Octane
2,6,10-Trimethyl-dodecane	3,5-Dimethyl-2-(3-methylbutyl)pyrazine		2,7-Dimethyl-octane
(+)-Sativen	4,8-Dimethyl-undecane		Methyl cinnamate
1,6-Dimethyl-naphthalene	2,4-Di-tert-butylphenol		Tetradecane
2,6,10-Trimethyltetradecane	1,2,3-Trimethyldiaziridine		(±)-β-Barbatene
	Dehydro-ar-ionene		3,3-Dimethyl-Hexane
			1-Octen-3-ol

## Data Availability

The original contributions presented in the study are included in the article; further inquiries can be directed to the corresponding author and the first author.
